# 612. Pharmacokinetics of Piperacillin-Tazobactam in Patients with Severe Infections

**DOI:** 10.1093/ofid/ofac492.664

**Published:** 2022-12-15

**Authors:** Ana S Rodríguez-Báez, Arturo Ortiz-Álvarez, Melissa Romano-Aguilar, Fidel Martínez-Gutiérrez, Silvia Romano-Moreno, Rosa C Milán-Segovia, Susanna Medellín-Garibay

**Affiliations:** Universidad Autónoma de San Luis Potosí, San Luis Potosi, San Luis Potosi, Mexico; Hospital Central “Dr. Ignacio Morones Prieto”, San Luis Potosi, San Luis Potosi, Mexico; Universidad Autónoma de San Luis Potosí, San Luis Potosi, San Luis Potosi, Mexico; Universidad Autónoma de San Luis Potosí, San Luis Potosi, San Luis Potosi, Mexico; Universidad Autónoma de San Luis Potosí, San Luis Potosí, San Luis Potosi, Mexico; Universidad Autónoma de San Luis Potosí, San Luis Potosí, San Luis Potosi, Mexico; Universidad Autónoma de San Luis Potosí, San Luis Potosí, San Luis Potosi, Mexico

## Abstract

**Background:**

Piperacillin-Tazobactam (PIP/TAZ) is a combination of a β-lactam antibiotic with a β-lactamase inhibitor, used to treat moderate to severe infections due to its broad-spectrum antibacterial activity. Its bactericidal effect is time-dependent. Therefore, the free-drug concentration should remain above the minimum inhibitory concentration during at least 50% of the dosing interval. Patients with severe infections develop pathophysiological changes that alter drugs pharmacokinetics (PK), leading to only 30% of probability of target attainment in clinical setting.

**Methods:**

A prospective observational study was performed in patients with severe infections from Hospital Central “Dr. Ignacio Morones Prieto”. The protocol was approved by the Research and Ethics Committee (register 05-20) and patients signed written informed consent. Samples were collected at steady-state and plasma concentrations were quantified by liquid chromatography coupled to mass spectrometry. Data were analyzed by a population approach using NONMEM® software.

**Results:**

A total of 52 patients were included (52% male) with a mean age of 46 ± 17 years and a body mass index of 25 ± 5 Kg/m^2^. According to the Akaike information criterion and visual inspection, a one-compartment open model was chosen to describe the concentration vs time data (n=156) for both drugs. Typical values (relative standard error) of PK parameters obtained were Clearance [CL_PIP_ (L/h)] = 8.79 (12%) and Volume of distribution [V_PIP_ (L)] = 17.6 (13%); and CL_TAZ_ (L/h) = 12.6 (14%), V_TAZ_ (L) = 32.8 (13%). Interindividual variability (IIV) of each parameter was modeled by exponential error and reported as coefficient of variation as follows: 75.3% and 88.7% for CL; 67.2% and 68.8% for V of PIP and TAZ, respectively. Finally, residual error was modeled as additive and presented a standard deviation (SD) of 7.28 µg/mL for PIP, and for TAZ was modeled as a combined with a SD of 0.22 µg/mL and a coefficient of variation of 17.32%.

**Conclusion:**

Individualization and optimization of β-lactam dosing are essential in drugs with wide IIV as PIP/TAZ; hence, development of a population PK model will provide a valuable aid in explaining and quantifying some of this variability to allow a priori predictions to design initial regimens to reach pharmacotherapeutic targets.

**Disclosures:**

**All Authors**: No reported disclosures.

